# Nectin-1-specific entry of herpes simplex virus 1 is sufficient for infection of the cornea and viral spread to the trigeminal ganglia

**Published:** 2012-11-16

**Authors:** Navika D. Shukla, Vaibhav Tiwari, Tibor Valyi-Nagy

**Affiliations:** 1 Department of Pathology, College of Medicine, University of Illinois at Chicago, Chicago, IL; 2 Illinois Mathematics and Science Academy, Aurora, IL; 3 Department of Microbiology and Immunology, Midwestern University, Downers Grove, IL

## Abstract

**Purpose:**

Primary and recurrent infections of the cornea by herpes simplex virus 1 (HSV-1) are important causes of eye disease. Three unrelated classes of glycoprotein D receptors for HSV-1 entry into cells have been identified. This study was undertaken to uncover the relative significance of nectin-1 as an entry receptor in corneal infection and HSV-1 spread to the trigeminal ganglia (TG), a site important for HSV-1 latency and recurrent corneal infection.

**Methods:**

To assess the significance of nectin-1, a member of the immunoglobulin superfamily, in primary HSV-1 infection and spread to the TG, we used a murine model of corneal infection and a HSV-1 mutant, KOS(Rid1), which can only use nectin-1 for entry. Immunohistochemistry, real-time PCR, and plaque assays using HSV-1 infected tissues were performed.

**Results:**

We demonstrated that receptor usage by HSV-1 limited to nectin-1 does not significantly change the spread of HSV-1 in the corneal epithelium during primary infection. We also found that nectin-1-specific entry does not affect the capacity of the virus to spread to the TG from the cornea.

**Conclusions:**

Our findings suggest that nectin-1 alone is sufficient for HSV-1 entry into the cornea and spread to the TG.

## Introduction

Herpes simplex virus 1 (HSV-1) can infect the human cornea and cause significant eye disease. During primary corneal HSV-1 infection, the virus enters cells and nerve endings in the corneal epithelium and spreads by axonal transport to the trigeminal ganglia (TG), a site important for HSV-1 latency and recurrent corneal infection [1].

HSV-1 entry into cells is initiated by specific interactions of viral envelope glycoproteins with host cell surface receptors [1]. The virus attachment to cells is mediated by glycoprotein B (gB) and/or glycoprotein C binding to cell surface heparan sulfate proteoglycans [2]. Binding of HSV-1 to heparan sulfate proteoglycans is followed by the binding of glycoprotein D (gD) to one of its receptors expressed on the host cell surface [3]. Thereafter, a multiprotein fusion complex involving gD, its receptor, three additional HSV glycoproteins, gB, glycoprotein H (gH), and glycoprotein L (gL), and possibly an additional gB coreceptor trigger penetration of the viral envelope with the plasma membrane of host cells [4]. As a result, viral capsids and tegument proteins are released into the cytoplasm of the host cell.

The gD receptors are represented by three structurally unrelated families of cell surface molecules. These include herpes virus entry mediator (HVEM), a member of the tumor necrosis factor receptor family [5]; nectin-1, which belongs to the immunoglobulin superfamily [6-8]; and a specifically modified form of heparan sulfate, 3-O-sulfated heparan sulfate (3-OS HS) [9]. HVEM mediates HSV-1 entry into human T lymphocytes and trabecular meshwork cells, and is expressed in many human tissues, including the lung, liver, kidney, and lymphoid tissues [5,10]. Nectin-1 mediates the entry of HSV-1 and HSV-2, and is extensively expressed by the cells of epithelial and neuronal origin [7,8,11,12]. The polysaccharide receptor 3-OS HS is expressed by multiple human cell lines (e.g., neuronal and fibroblasts) and mediates entry of HSV-1 but not HSV-2 [9,13]. Very limited information is available on the relative importance of individual gD receptors in HSV-1 entry. Nectin-1 and HVEM have been shown to be important for HSV-2 entry and spread in vivo and the same has been demonstrated for HSV-1 using receptor knockout mice [14,15]. Here, we use an alternative approach, examining a mutant virus, HSV-1(KOS)Rid1, which has a point mutation in gD that renders the virus unable to use HVEM or 3-OS HS [1,16]. Using HSV-1(KOS)Rid1, which only uses nectin-1 for entry and a murine model of corneal infection, we demonstrate that nectin-1 may be sufficient to allow virus infection of the eye and spread of the virus to the TG.

## Methods

### Viruses and cells

Reporter strains of wild-type HSV-1(KOS) were used. KOS-tk12 and KOS-Rid1-tk12 express β-galactosidase under control of the HSV-1 infected cell protein 4 (ICP4) promoter. The virus strains were propagated and tittered on Vero cells.

### Animals and tissue processing

All animal handling and experiments were performed according to the institutional animal care and use guidelines and adhered to the Association for Research in Vision and Ophthalmology (ARVO) statement for the Use of Animals in Ophthalmic and Vision Research. Four- to six-week-old inbred BALB/c mice (Harlan Laboratory, Indianapolis, IN) were used in this study. Mice were inoculated on their left corneas with either 1×10^5^ plaque forming units of a recombinant HSV-1 mutant; nectin-1-specific strain KOS-rid-1-tk12; or its wild-type counterpart, KOS-tk12, as a control [17]. Virus inoculation was performed after corneal scarification with a hypodermic needle. During the inoculation, the animals were kept under ketamine/xalazine anesthesia. Animals were monitored every day, and none of the animals showed any neurological symptoms of the disease or died during the experiments. Since our experiments were designed to study the primary infection, the animals were euthanized by CO_2_ inhalation at 2 and 5 days after inoculation. Groups of uninfected, normal age-matched mice were also euthanized for control purposes. The left eyeballs and TG were aseptically removed and were either processed for formalin fixation, paraffin embedding, sectioning, and histological and immunohistochemical studies to detect HSV-1 protein expression by immunohistochemistry, or snap frozen in liquid nitrogen for virus titration or PCR studies to detect HSV-1 DNA.

### Quantitative real-time polymerase chain reaction

Real-time quantitative PCR was performed in PCR tubes using DNA prepared according to the manufacturer’s protocol (QIAamp DNA Mini kit; Qiagen Inc., Valencia, CA) using a Smart Cycler System (Cepheid). From each infection group, five TGs were pooled together and processed for the detection of HSV-1 DNA by real-time PCR. Primers against HSV-1 (KOS) gD were designed using Clone Manager 6 program (Sci Ed Central). The forward primer included the sequence AAG ACC TTC CGG TCC TG and the backward, TCC AAC ACG GCG TAG TA. The reaction included 50 cycles under the following conditions: denaturation at 95 °C for 30 s, annealing at 58 °C for 10 s, and extension at 72 °C for 16 s. After amplification, one cycle of melting curve from 60 to 95 °C with a transition rate of 0.2 °C/s and continuous detection of fluorescence was performed [18].

### Immunohistochemistry

Five animals for each infection group were processed for immunohistochemistry, which was performed as described previously [19]. Briefly, cut tissue sections for immunohistochemistry were deparaffinized with xylene and rehydrated through a series of graded ethanol. Tissue sections were then hydrated with distilled water, and antigen retrieval was performed using Target Retrieval Solution (10× Concentrate; Dako, Carpinteria, CA). Nonspecific staining was blocked using H_2_O_2_ for 10 min, followed by protein block with fetal bovine serum (Sigma) for 10 min. Sections were incubated with polyclonal antibodies against HSV-1 (Abcam Inc.) followed by detection with horseradish peroxide–conjugated secondary antibodies and a substrate yielding an insoluble (brown) product (Dako, Carpinteria, CA) [19-21].

### Plaque assay

Viral replication in the ocular globes was analyzed by plaque assay. Five ocular globes of each kind were pooled, homogenized, and processed for the plaque assay. Briefly, monolayers of Vero cells were plated in 24-well tissue culture dishes and 10-fold serial dilutions of the homogenized tissues resuspended in PBS were added for plaque formation. Cells were incubated at 37 °C and harvested at 48 h post infection. At the time of the harvest, cells were washed, fixed, and stained with Giemsa stain. Infectivity was measured by the number of plaque-forming units. Plaques were quantitated under a microscope (Axiovert 100 M; Zeiss).

### Statistical analysis

The statistical significance of the data were analyzed by the Student *t* test and Fisher’s exact test, using Instat (GraphPad, San Diego, CA). Results were considered statistically significant when the p value was <0.05. Statistical analysis was used for the plaque formation results discussed in Table 1.

**Table 1:** Quantification of infectious HSV-1 in the ocular globes of BALB/C mice following mice inoculation of KOS-tk12 or KOS-rid1-tk12.

Inoculum	HSV-1 titers* (PFU) at	
	2 days p.i.	5 days p.i.
KOS-tk12	3.6×10^4^	9.5×10^1^
KOS-rid-tk12	2.9×10^4^	8.8×10^1^
Uninfected	0	0

*For each time point and treatment group, 5 ocular globes were pooled, homogenized, and titered in triplicate cultures.

## Results

### Nectin-1 alone may be sufficient for herpes simplex virus 1 entry into the cornea

**Figure 1:**
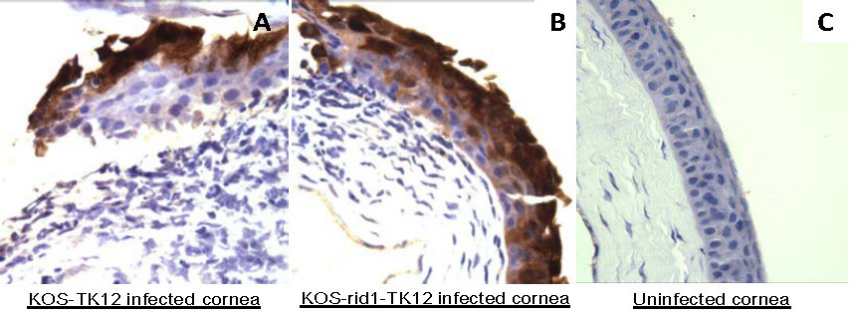
Histopathological changes and the expression of herpes simplex virus 1 proteins in the corneas of BALB/c mice. Formalin-fixed, paraffin-embedded, murine ocular tissues were sectioned and stained with a rabbit antiserum raised against herpes simplex virus 1 (HSV-1). The brown staining indicates HSV-1 proteins. Corneas 2 days after virus inoculation are shown in panel (**A**) KOS-tk12 and panel (**B**) KOS-Rid1-tk12. Uninfected cornea section is shown in panel (**C**) Representative pictures are shown.

To determine the sole contribution that nectin-1 makes in HSV-1 infection of the eye, groups of female BALB/c mice were inoculated with a recombinant nectin-1-dependent HSV-1 strain KOS-rid1-tk12 or its wild-type counterpart, KOS-tk12, as a control [17]. The KOS-rid1-tk12 virus relies solely on nectin-1 and does not use any other known gD receptors for entry [1]. During infection with the two viruses, none of the animals showed any neurological symptoms of the disease or died during the experiments. However, sections of the corneas from KOS-tk12 or KOS-rid-1-tk12 inoculated mice euthanized at 2 and 5 days after virus inoculation showed signs of inflammation and expression of HSV-1 proteins in the corneal epithelium (Figure 1B,C). Extensive corneal inflammation and widespread HSV-1 protein expression in the corneal epithelium was detected in four out of five KOS-tk12 inoculated mice, and three out of five KOS-rid-1-tk12 inoculated mice at 2 days after virus inoculation. Less severe inflammation and only marginal HSV-1 protein expression in the corneal epithelium was detected in one out of five KOS-tk12-inoculated mice, and two out of five KOS-rid-1-tk12-inoculated mice at 5 days after virus inoculation. Ocular tissues derived from uninfected mice showed no evidence of inflammation or HSV-1 protein expression (Figure 1C).

### Nectin-1-specific virus results in titers similar to the wild-type virus infection of the cornea

The snap frozen eyeballs from the infected animals and uninfected controls were processed for determination of the presence of infectious HSV-1 by pooling and homogenizing five eyeballs for infecting monolayers of Vero cells to determine the virus titers in the homogenates. As shown in Table 1, there were only marginally fewer HSV-1 KOS-rid-1-tk12 titers in Vero cells than the control, KOS-tk12 titers, at both 2 days and 5 days after inoculation. The difference at 2 days post infection reached statistical significance, and it is likely that lack of ability to use HVEM and/or 3-OS HS may marginally affect the capacity of the virus to cause infection in the eye. However, in terms of the disease symptoms, we did not notice any significant differences. Eyeballs derived from uninfected control mice contained no infectious virus.

### Use of nectin-1 may be sufficient for the virus to spread to the trigeminal ganglia

**Figure 2:**
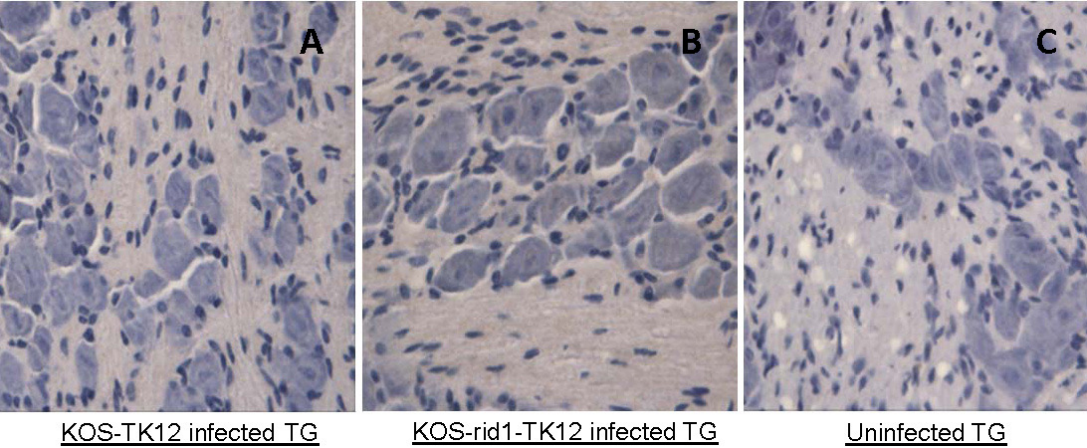
Expression of herpes simplex virus 1 proteins in the trigeminal ganglia of BALB/c mice. Five days after virus inoculation, murine trigeminal ganglia (TG) were isolated, formalin fixed, paraffin embedded, sectioned and stained with a rabbit antiserum raised against herpes simplex virus 1 (HSV-1). A lack of brown staining indicates lack of HSV-1 proteins. TG tissue 5 days after KOS-tk12 inoculation is shown in panel A and TG 5 days after KOS-Rid1-tk12 inoculation is shown in panel **B** Uninfected TG section is shown in panel **C** HSV-1 infected corneas were used as positive control (Figure 1). Representative pictures are shown.

**Figure 3:**
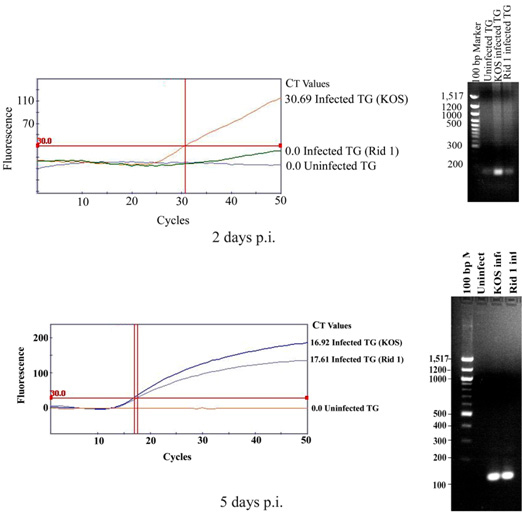
Detection of herpes simplex virus 1 DNA in the trigeminal ganglia. Five left trigeminal ganglia (TG) from each treatment group were pooled and processed for the detection of herpes simplex virus 1 (HSV-1) DNA by PCR. The tissues were homogenized, DNA extracted, and amplified using real-time PCR. HSV-1 DNA (at a threshold cycle, CT, of around 15) was detected in trigeminal tissues derived from KOS-tk12 and KOS(Rid1)-tk12 inoculated animals. CT values represent the fractional cycle number at which fluorescence passes a fixed threshold (indicated). The tissues from mock-infected animals were negative.

Next, we decided to measure the presence of HSV-1 DNA in the TG of animals infected with the mutant virus and the control. The TG derived from KOS-tk12- or KOS-rid-1-tk12-inoculated mice euthanized 2 and 5 days after virus inoculation showed no evidence of inflammation; no immunohistochemical evidence of HSV-1 protein expression was detected [22-24] (Figure 2A-C). To determine the presence of viral DNA in the TG, five left TGs from each treatment group were pooled and processed for the identification of HSV-1 DNA by real-time PCR (Figure 3). As shown in Figure 3, at 2 days post infection, KOS-tk12 DNA was detected much earlier (at threshold cycle, CT, of around 30) than KOS-Rid1-tk12 and the amount of product seen on an agarose gel was also higher for KOS-tk12 than the product obtained from KOS-Rid1-tk12 replication during the same time. This result would suggest that KOS-Rid1-tk12 reached the TG with some delay relative to KOS-tk12 from the cornea possibly due to less efficient entry of KOS-Rid1-tk12 into nerve endings in the cornea or less efficient axonal spread from the cornea to the TG. DNA curves from uninfected animals were negative. However, at 5 days post infection, virtually no differences were seen in detection of HSV-1 KOS-tk12 and KOS(Rid1)-tk12 DNAs (at a CT of around 17) in tissues derived from the infected animals (Figure 3). These results indicate that both KOS-tk12 and KOS-rid-1-tk12 replicated in the cornea following corneal inoculation and spread to the TG.

## Discussion

Our findings suggest that receptor usage limited to nectin-1 does not significantly change the replication and spread of HSV-1 in the corneal epithelium during the early stages of the primary infection, and does not affect the capacity of the virus to spread to the TG. However, as KOS-Rid1-tk12 demonstrated a minor impairment in ocular replication and minor delay in its spread to the TG, our study cannot rule out the possibility of a role of entry receptors other than nectin-1 in the pathogenesis of the early stages of primary corneal HSV-1 infection. It is also important to emphasize that other entry coreceptors such as heparan sulfate that binds gB or gC and some other newly discovered receptors for gB such as non-muscle myosin heavy chain-IIA (NMHC-IIA), myelin-associated glycoprotein (MAG), paired immunoglobulin-like type 2 receptor (PILR-α), and gH (integrins) may still be important, and our study is limited to demonstrating the importance of a gD receptor in HSV-1 entry into the cornea [4]. The Rid1 mutant virus that we used has been shown to have a key point mutation in gD [1]; therefore, its ability to use any gB or gH coreceptors is not expected to differ significantly from the wild-type virus. However, studies will be designed in the future to determine whether wild-type HSV-1 differs from the Rid1 virus in its ability to use gB and gH coreceptors. Likewise, the use of nectin-2 by the mutant virus may compensate in part for the inability to use HVEM or 3-OS HS. However, nectin-1 is the most relevant receptor for ocular infection, since wild-type HSV-1 fails to use nectin-2 [1]. In support of our findings, nectin-1 knockout studies have demonstrated the loss of corneal infection by HSV-1 [14]. Further studies using antibodies or RNA interference technology may be conducted in the future to further verify the role of nectin-1 in vivo.

Our findings are especially important given the serious manifestations of HSV-1 in the eye, including the possibility of latency in the cornea [25-27]. In any case, the virus is a leading infectious cause of blindness in developed countries [28], and very little can be done at present to stop recurrent problems associated with herpetic keratitis [29]. Nectin-1 is highly expressed in the cornea [20]. Our demonstration of the significance of nectin-1 during ocular infection, combined with similar findings using knockout mice [14], is sure to spark new strategies and the development of novel agents to treat ocular infection. Currently, entry-based inhibitors do not exist for the treatment of ocular herpes. The only drug suspected of blocking spread, docosanol (Abreva^TM^), shows efficacy in reducing symptoms, but it has not been approved for ocular applications [30]. Thus, studying HSV-1 entry remains an exciting area for drug development, and our study paves the way for targeting nectin-1/gD interaction for new preventive and therapeutic treatments for HSV-1-induced ocular disease [31]. In addition to ocular disease, the observations reported here may also help in the development of novel strategies against HSV-1 infections of the nervous system, another clinically highly significant target of HSV-1 [32,33].
